# A novel assessment of fine-motor function reveals early hindlimb and detectable forelimb deficits in an experimental model of ALS

**DOI:** 10.1038/s41598-022-20333-1

**Published:** 2022-10-11

**Authors:** C. Sahara Khademullah, Yves De Koninck

**Affiliations:** CERVO Brain Research Centre, Université Laval, 2601 Chemin de la Canardière, Quebec, QC G1J 2G3 Canada

**Keywords:** Motor control, Amyotrophic lateral sclerosis

## Abstract

Amyotrophic lateral sclerosis (ALS) is a neurodegenerative disorder associated with the loss of cortical and spinal motor neurons (MNs) and muscle degeneration (Kiernan et al. in Lancet 377:942–955, 2011). In the preclinical setting, functional tests that can detect early changes in motor function in rodent models of ALS are critical to understanding the etiology of the disease and treatment development. Here, we established a string-pulling paradigm that can detect forelimb and hindlimb motor deficits in the SOD1 mouse model of ALS earlier than traditional motor performance tasks. Additionally, our findings indicate that early loss of forelimb and hindlimb function is correlated with cortical and spinal MN loss, respectively. This task is not only ecological, low-cost, efficient, and non-onerous, it also requires little animal handling and reduces the stress placed on the animal. It has long been a concern in the field that the SOD1 mouse does not display forelimb motor deficits and does not give researchers a complete picture of the disease. Here, we provide evidence that the SOD1 model does in fact develop early forelimb motor deficits due to the task’s ability to assess fine-motor function, reconciling this model with the various clinical presentation of ALS. Taken together, the string-pulling paradigm may provide novel insights into the pathogenesis of ALS, offer nuanced evaluation of prospective treatments, and has high translational potential to the clinic.

## Introduction

Amyotrophic lateral sclerosis (ALS) is a fatal neurodegenerative disease that belongs to a broad group of disorders known as motor neuron diseases (MNDs). This disease is characterized by catastrophic muscle weakness and atrophy that eventually spreads across the body as it progresses into later stages^1^. Following the initial onset of symptoms, approximately 50% of cases are fatal within the first 1.5 years, typically resulting from respiratory failure^1^. More than 90% of ALS diagnoses can be classified as sporadic (sALS), as their cause is largely unknown; the remaining 10% of cases fall into the category of familial ALS (fALS)^2^. Of the fALS cases, 25% result from mutations on chromosome 21 in the gene that encodes the copper (Cu)-zinc (Zn) superoxide dismutase 1 (SOD1) enzyme. The toxic gain-of-function mutation also contributes to approximately 5% of sALS diagnoses^3^. The SOD1*G93A mouse model is the most widely used model in ALS research to date because of its ability to closely mimic the clinical phenotype of human ALS^4^. This model has come under criticism for it’s apparent lack of cortical involvement and relatively limited onset of forelimb dysfunction; however, recent evidence has emerged indicating that the cortex plays a larger role than initially acknowledged^5–8^. Additionally, unlike the string-pulling paradigm we propose here, current motor-performance tasks used to assess symptoms associated with ALS, such as grip strength and the rotarod, commonly measure gross motor function and, thus, cannot be considered adequately sensitive to detect fine-motor changes associated with forelimb and/or early hindlimbs deficits.

SOD1*G93A mice are typically considered pre-symptomatic between 8–12 weeks, at which point there is clear evidence of cortical dysfunction^7,9^. Following this, gradual weight loss is evident, and through the use of classical gross motor performance tests sensitive to hindlimb dysfunction, including the rotarod test, gait analysis, and hanging wire tests, a decline in hindlimb function has been reported typically at 14 weeks^9^. Each of the aforementioned tasks have significant limitations and are typically financially expensive and time-consuming, requiring significant stress-inducing handling and training with multiple data collection sessions per week across multiple trials^9,10^. The rotarod is the most widely used test to measure overt hindlimb function, balance, and coordination. While this task is often plagued with performance-based limitations including animals clinging to the rod and rotating with it, and the refusal to perform the task, it continues to be the gold-standard for detecting the onset of ALS pathology in rodent models of the disease^11^. When tested early during the asymptomatic/pre-symptomatic phases, Mancuso et al.^12^ reported that SOD1 mice showed a failure to perform on the rotarod as early as 12 weeks, while other groups have reported a steady decline in performance when compared to their non-transgenic littermates between 14–16 weeks^7,9,13–16^. This discrepancy in the literature can be attributed to the various ramp protocols used when administering the task and the different definitions of clinical onset used (e.g., first failure to perform or significant divergence from WT performance), which has lead to concerns regarding the lack of reproducibility and consistency for this task^17^. Gait analysis, while often considered an inconsistent measure and onerous for the experimenter, can detect subtle differences in gait, stance and stride as early as 11 weeks in SOD1 mice when compared to non-transgenic littermates^11,18,19^. Grip strength measurements are commonly employed to determine the extent of forelimb muscle weakness as the disease progresses. Results from this test have established a reduction in the maximum peak force at ~ 14 weeks in SOD1 mice when compared to their non-transgenic littermates^7,20^. However, this task is largely criticized for having mixed results due to the test’s dependence on the force applied by the experimenter^9,17^. Finally, the hanging wire can detect the onset of pathological motor symptoms in the SOD1 model between 14–16 weeks^7,9,19,21,22^. Similar to the rotarod, clinical symptom onset is determined by reporting the animal’s first failure to perform the task or by determining the point at which SOD1 mice significantly deviate from their non-transgenic littermates. This tasks is frequently confounded by the willingness of the animal to perform the stressful task and the tendency for the rodent to use the walls on either side of the wire as support^11^.

**Figure 1:**
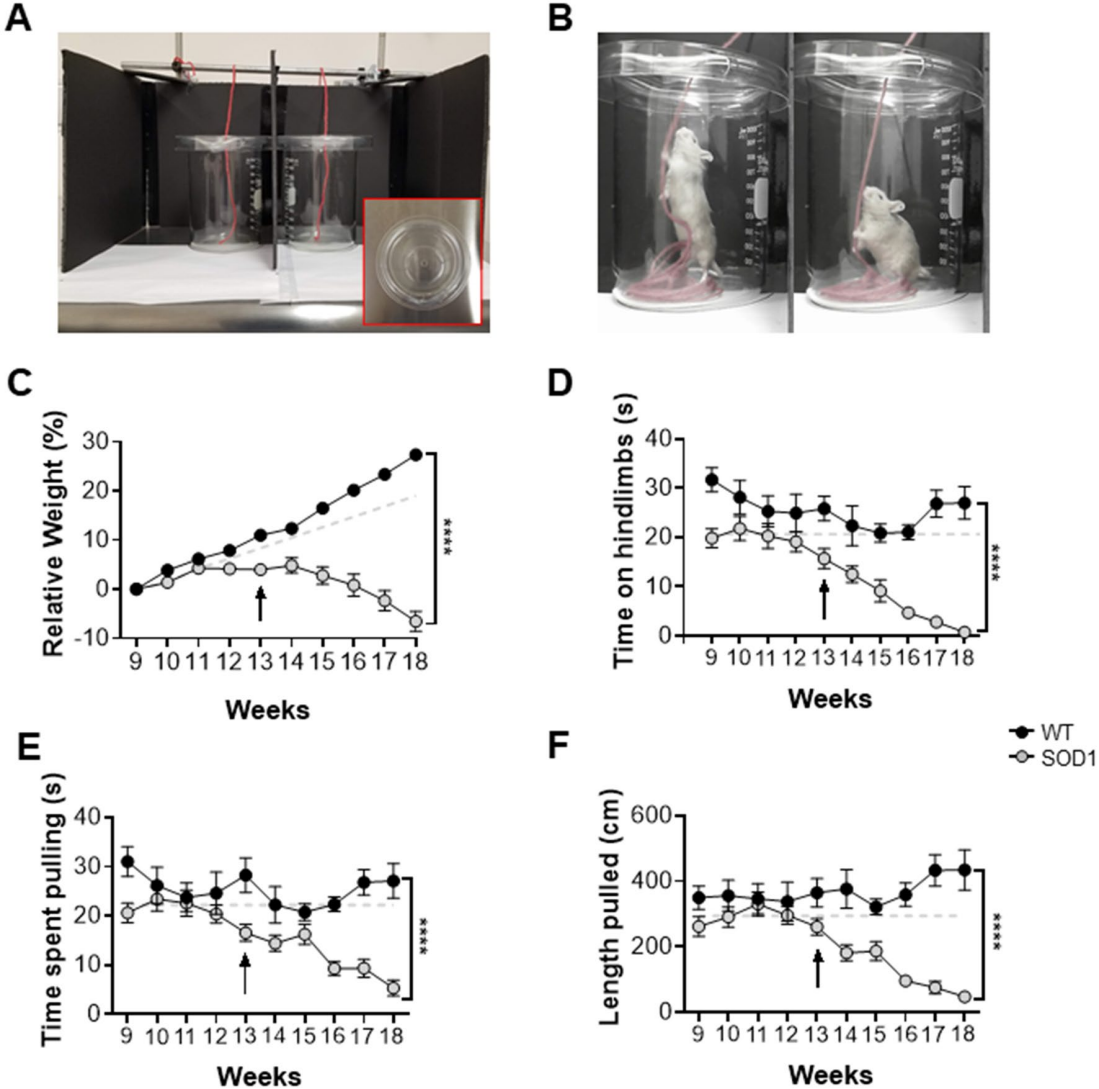
String-pulling task can detect early deficits in fine forelimb motor function in the SOD1 mouse. (**A**) Complete setup with two recording chambers and inserted string. Inset shows a recording chamber with plastic tubing attached to the lip and lid on top. (**B**) Example image of a WT mouse performing string-pulling task in the act of pulling while standing on hindlimbs (left) and the same mouse pulling while in the seated position. (**C**) Weekly weight measurements were reported as relative weight change to week 9. (**D**–**F**) Parameters of string-pulling task separate into; amount of time spent off of seat and up onto hindlimbs, amount of time spent pulling the string and length of string pulled for WT (n = 16; eight males, eight females) and SOD1 littermates (n = 24; 12 males, and 12 females). Weight and behavior was quantified using a mixed-effect analysis Sidak's multiple comparisons test and plotted as weekly averages with a z score of 0.5 set as the threshold from the baseline (grey dotted line). Error bars denote SEM.

**Figure 2:**
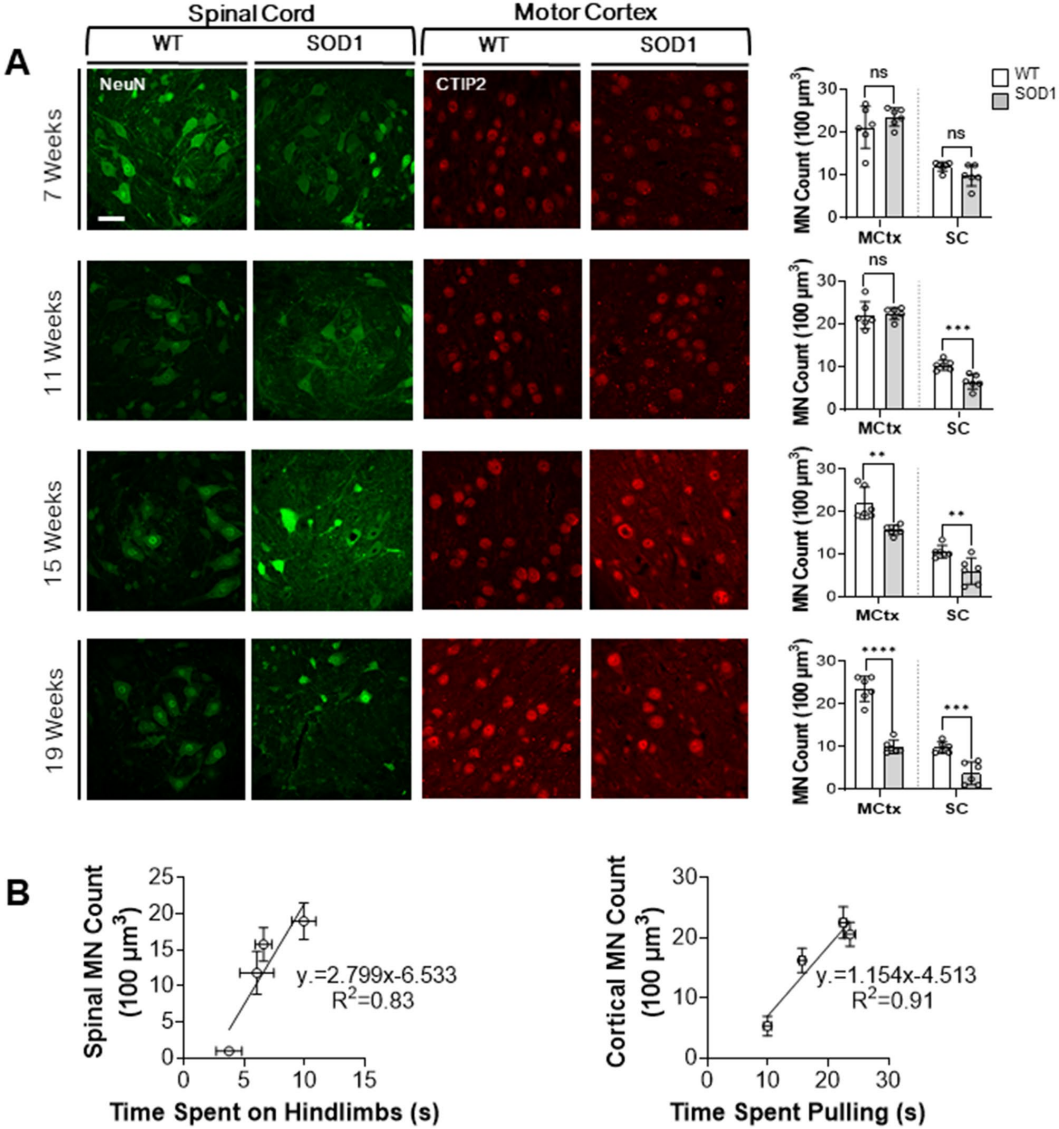
Loss of forelimb and hindlimb function is associated with cortical and spinal MN loss in the SOD1 mouse. (**A**) Representative confocal images showing spinal MN (NeuN^+^ neurons > 250 µm) and cortical MN counts (CTIP2^+^ neurons) at weeks 7, 11, 15 and 19. Scale bar = 50 µm. Scatterplot of MN counts were combined for both the motor cortex and spinal cord. Cell counts were made from multiple slices from the same animal and then averaged and reported as one n-value; n = 6 for all groups. All images were acquired from male mice. Unpaired *t* test. (**B**) Scatter plots indicating loss of MNs in the spinal cord and motor cortex is associated with decreased time spent on hindlimbs (right) and pulling (left) respectively.

Here, we conducted weekly weight measurements and found that SOD1 mice began to detectably lose weight relative to their non-transgenic littermates at 13 weeks (Fig. 1C). Using our string-pulling paradigm, we were able to also detect a decline in hindlimb function as early as 13 weeks (Fig. 1D; Supp. Fig. 1, Supp. Table 1). This decline was consistent with a significant loss of MNs in the ventral horn in the L5 region of the spinal cord (Week 11: P < 0.001) (Fig. 2A). Based on these findings we can conclude that the string-pulling task is sensitive to early loss of hindlimb function in the SOD1 mice, earlier than typically reported with the rotarod test and other motor performance tests sensitive to hindlimb dysfunction.

The SOD1 mouse model has largely been regarded as a model of hindlimb-onset ALS^23^. Spinal MNs appear to be selectively vulnerable in patients and rodents with the SOD1 mutation alike for reasons that are not well known^24,25^. As a result, potential therapeutics focused on the fate of cortical MNs in this model are often overlooked. Recently, however, evidence from the SOD1 model has revealed that cortical MNs are in fact prone to dysfunction and cell death which may ultimately lead to forelimb dysfunction^7,26^. Additionally, SOD1 mice exhibit both spinal and cortical astrogliosis, deficits associated with axonal transport and mitochondrial vacuolization, and abnormal formation of neurofilaments^27,28^. Provided that ALS affects numerous sections of the corticospinal tract throughout its progression, it is necessary to find sensitive measures that can detect these changes as they occur. Grip-strength measurements are commonly employed to determine the extent of forelimb muscle weakness as the disease progresses. Results from this test often have a high degree of heterogeneity making it prone to bias due to the test’s dependence on the applied degree of force from the experimenter^9,17^. Weekly performances on the string-pulling task revealed detectable decrease in the time spent pulling and length pulled in SOD1 mice compared to their non-transgenic littermates beginning as early as 13 weeks (Fig. 1E,F; Supp. Table 1, Supp. Fig. 2). Interestingly, significant cortical MN loss in the L5-M1 was significantly correlated with the onset of the decline observed in forelimb function (Week 15: P < 0.01) (Fig. 2A). No significant differences were observed between the two sexes (Supp. Fig. 1A–C). Interestingly, cortical disturbances and MN loss in this region has been observed at similar timepoints in previous reports^5,7^. Cortical MN loss was also significantly correlated with forelimb (R^2^ = 0.91) and hindlimb (R^2^ = 0.94) deficits (Fig. 2B, left), while spinal MN loss was correlated with hindlimb deficits (R^2^ = 0.83) (Fig. 2B, right). While the literature is inconclusive, our results are in line with rodent lesion and optogenetic studies that show manipulating the motor cortex can indeed result in altered forelimb function^29–31^. Results collected with the string-pulling task demonstrates, for the first time, that early fine-motor forelimb deficits can be detected in the SOD1 mouse around the same timepoint as hindlimb deficits, reconciling the SOD1 model with the clinical phenotype of ALS.

In conclusion, we have identified the string-pulling task as a novel fine-motor performance test that can be utilized in ALS mouse models to evaluate subtle changes in both forelimb and hindlimb function associated with degeneration of the vulnerable cortical and spinal MN populations in the same trial earlier than any other classically used motor performance test. Cortical function is critical for fine-motor function. Thus, to detect cortical MN disturbances, a fine-motor skill test, such as the string-pulling task proposed here, is necessary. Here, we show for the first time, that the SOD1*G93A mouse displays both hindlimb and fine-forelimb motor deficits as early as 13 weeks. Not only does this fine-motor performance task have the advantage of allowing for more precise pre-clinical treatment outcome measurements, it is also minimally invasive and requires little animal handling and no training. Moreover, these findings serve to fulfill the unmet need of investigating cortical and forelimb motor deficits in the SOD1 mouse model that the field has long been faced with. A challenge that has left the field with a void when studying the various clinical manifestations of ALS (upper versus lower motor onset). Furthermore, in addition to the test’s holistic nature, it can also yield a more sensitive detection of both forelimb and hindlimb dysfunction in a single trial and can easily be applied to other rodent disease models and adapted to assess various motor-performance outputs. Additionally, due to the task’s ecological nature, it does not require any food or water deprivation, rather motivation to perform is simply provided by means of adding an appetizing reward to the top of the testing apparatus. Unlike the commonly employed grip-strength test, in which the experimenter can introduced variability in the stimulus, this task is completely experimenter independent. The lack of food/water deprivation, minimal handling time, and minimal stress resulted in 100% of motivated performances, making this test highly reliable and efficient. Increasing the reliability and efficiency of behavioural tests is paramount to reducing the number of experimental animals used. Due to the test’s inexpensive price point, both in terms of equipment and analysis tools, it is easily accessible to all researchers. While the present analysis was performed manually at this time, development of an automated system is currently underway. Finally, because this task incorporates both global and fine motor-skill components, it has a high translational potential to the clinic. As a result, it can offer detailed evaluations of potential therapeutics and provide novel insights into the pathogenesis of ALS.

## Materials and methods

### Animals

All mice were obtained directly from Jackson Laboratories: SOD1*G93A (B6SJL-Tg(SOD1*G93A)1Gur/J). Non-transgenic (WT) mice were litter-matched, and age-matched. For all behavioural experiments, an equal proportion of female and male mice were used. Mice were randomly assigned to experimental groups. Mice were housed under a 12-h/12-h light/dark cycle with ad libitum access to food and water and were randomly allocated to different test groups. All experiments were approved by Université Laval’s Animal Care Committee and are in accordance with the guidelines of the Canadian Council on Animal Care. Additionally, this study is reported in accordance with the ARRIVE guidelines.

### String-pulling task

String-pulling tests were performed twice weekly between weeks 9–18. Weight measurements were taken weekly to evaluate loss of body weight and muscle mass pertaining to disease progression.

Mice were habituated to testing room in their home cages for at least 1 h. Mice were then placed into the testing apparatus which was a transparent modified glass beaker (1L, Pyrex) with 5/16 in flexible vinyl tubing (Versilon, cat. # 26515138) around the lip to prevent the lid from moving (Fig. 1A). A transparent plastic lid with a 1 cm diameter hole was then placed on top of the testing apparatus and a red string (5 mm) tied to a scaffold above was lowered into the apparatus. To eliminate distraction, 3 walls (rear and sides, leaving the top uncovered, each wall; 10 × 10 × 10) were assembled out of black foam board around the apparatus (Fig. 1A). A chocolate chip piece (Hersey’s Chipits) was placed on the lid near the hole as an incentive. Mice were allowed a 5 min habituation period in the beaker with the string placed inside prior to recording. Recordings were made with a Microsoft^®^ LifeCam Cinema Camera which was placed perpendicular (15 cm away from recording table) to the rear foam board wall. Recordings were made at a frame rate of 30 frames/s (1280 × 720) for 1 min beginning at the moment of first engagement with the string following the 5 min habituation period in order to prevent performance fatigue. After testing, mice were returned to their home cages.

### Forelimb assessment

The length of the string pulled during the 1 min recording was documented in cm. To quantitatively assess forelimb function, time (s) was recorded for the period which the mouse was griping onto the string with either one or both paws and engaged in a pulling behaviour (string movement towards the floor of the apparatus) (Fig. 1B).

### Hindlimb assessment

To assess hindlimb function, time was recorded for the period during which the mouse was griping the string with either one or both paws while in standing position up off its seat and onto its hind paws. Time spent pulling the string while in the seated position was recorded as time spent pulling, but not time spent on hindlimbs (Fig. 1B). The task was performed for two sessions per week with each session consisting of one trial to prevent performance fatigue. The average performance for each week was then calculated.

### Fluorescent immunolabelling

Mice were anesthetized under 4% isoflurane and transcardially perfused with ice-cold 1 × PBS, followed by 4% PFA. Brains and spinal cords were extracted and post-fixed in 4% PFA for 16 h at 4 °C. Spinal cords were cryoprotected in 30% sucrose and stored at − 80 °C. Brain slices containing the M1 were cut coronally at 40 µm thickness on a vibratome (Lecia VT1200S). Transverse sections were cut at a thickness of 30 µm from the L5 of the spinal cord with a microtome (Lecia SM2000 R). Free-floating sections containing the M1 and/or the L5 of the spinal cord were rinsed once in 1 × PBS for 5 min, followed by two more washes in 1 × PBS with 0.1% Triton-X. Slices were then blocked in 1 × PBS containing 10% goat serum and 0.1% Triton X-100 for 1 h at room temperature, followed by a 16-h incubation in 1 × PBS with 0.1% Triton-X-100 with mouse monoclonal anti-NeuN (1:500; EMD Millipore, MAB377) and/or rabbit polyclonal anti-CTIP2 (1:100, Abcam, ab28448) at 4 °C. Finally, slices were incubated in AlexaFluor488-conjugated goat anti-rabbit antibody for CTIP2 and AlexaFluor488-conguated Goat anti-mouse for NeuN for 2 h at room temperature. Mounted slices were imaged using a LSM 700 inverted confocal microscope (Zeiss). Images were acquired using the Zen Blue analysis software (Zeiss). Images were captured at 40X using a z-stack imaging method. In all cases, L5-M1 and the L5-ventral horn were identified with the 10 × objective, and the individual region of interest was chosen at random (experimenter was blinded) with the 40X objective. Motor neurons in the L5-M1 were identified as having CTIP2^+^ expression and counted manually in ImageJ. In the ventral horn, NeuN^+^ cell bodies > 250 µm in size were considered to be motor neurons and counted in ImageJ on a maximum intensity project image (previously described by Dutta et al.^32^). Imaging experiments were performed and analyzed in a blinded manner.

### Statistics

All data are presented as mean ± s.e.m., and each datum point represents an individual animal or experiment. Tests of statistical difference were performed with GraphPad Prism 9 software using unpaired *t* test (two-sided). Weight and behavioral measurements were analyzed using a mixed-effect analysis multiple comparisons test with a Sidak post-hoc test. Time course behavioural experiments were analyzed by averaging scores from SOD1 transgenic mice between weeks 9 and 11 which served as the baseline and calculating the deviation (z score) of the remaining SOD1 scores. Similarly, the average SOD1 weight measurements were taken between week’s 9–11 and extrapolated to week 18, which served as the baseline and showed the increase in weight the SOD1 mutant mice would have gained if they were to follow the trend of their non-transgenic littermates. Disease onset was defined as the first point when an animal deviated 20% from its own baseline. A z-score threshold of − 0.5 (20% deviation from the experimental group’s own baseline) was set to determine statistically significance between WT and SOD1 mice. Data used for statistical comparison fit a Gaussian (normal) distribution, and variances were equivalent between groups. For all experiments, a criterion α level was set at 0.05. For immunofluorescence experiments, cell counts were made from multiple slices from the same animal, and these were averaged and reported as one *n* value. To provide a meaningful value for motor-neuron counts, the number of cells counted in a field of view was divided by the area (1024 × 1024) and then multiplied by 1,000,000. Animals were excluded from analysis if they died before showing typical and predicted disease progression in accordance with the JAX ALS Mouse Manual.

## Supplementary Information

Supplementary Information.

## Data Availability

The datasets used and/or analysed during the current study available from the corresponding author upon reasonable request.

## References

[1] Kiernan MC (2011). Amyotrophic lateral sclerosis. Lancet.

[2] Renton AE, Chiò A, Traynor BJ (2013). State of play in amyotrophic lateral sclerosis genetics. Nat. Neurosci..

[3] Karch CM, Prudencio M, Winkler DD, Hart PJ, Borchelt DR (2009). Role of mutant SOD1 disulfide oxidation and aggregation in the pathogenesis of familial ALS. Proc. Natl. Acad. Sci. U.S.A..

[4] Shibata N (2001). Transgenic mouse model for familial amyotrophic lateral sclerosis with superoxide dismutase-1 mutation. Neuropathology.

[5] Ozdinler PH (2011). Corticospinal motor neurons and related subcerebral projection neurons undergo early and specific neurodegeneration in hSOD1G^93^A transgenic ALS mice. J. Neurosci..

[6] Clark RM, Brizuela M, Blizzard CA, Dickson TC (2018). Reduced excitability and increased neurite complexity of cortical interneurons in a familial mouse model of amyotrophic lateral sclerosis. Front. Cell. Neurosci..

[7] Khademullah CS (2020). Cortical interneuron-mediated inhibition delays the onset of amyotrophic lateral sclerosis. Brain.

[8] Marques C, Burg T, Scekic-Zahirovic J, Fischer M, Rouaux C (2021). Upper and lower motor neuron degenerations are somatotopically related and temporally ordered in the sod1 mouse model of amyotrophic lateral sclerosis. Brain Sci..

[9] Oliván S (2015). Comparative study of behavioural tests in the SOD1G93A mouse model of amyotrophic lateral sclerosis. Exp. Anim..

[10] Knippenberg S, Thau N, Dengler R, Petri S (2010). Significance of behavioural tests in a transgenic mouse model of amyotrophic lateral sclerosis (ALS). Behav. Brain Res..

[11] Brooks SP, Dunnett SB (2009). Tests to assess motor phenotype in mice: A user’s guide. Nat. Rev. Neurosci..

[12] Mancuso R (2014). Resveratrol improves motoneuron function and extends survival in SOD1G93A ALS mice. Neurotherapeutics.

[13] Hayworth CR, Gonzalez-Lima F (2009). Pre-symptomatic detection of chronic motor deficits and genotype prediction in congenic B6.SOD1G93A ALS mouse model. Neuroscience.

[14] Smittkamp SE, Brown JW, Stanford JA (2008). Time-course and characterization of orolingual motor deficits in B6SJL-Tg(SOD1-G93A)1Gur/J mice. Neuroscience.

[15] Miana-Mena FJ (2005). Optimal methods to characterize the G93A mouse model of ALS. Amyotroph. Lateral Scler. Neuron Disord..

[16] Weydt P, Hong SY, Kliot M, Möller T (2003). Assessing disease onset and progression in the SOD1 mouse model of ALS. NeuroReport.

[17] Mandillo S (2008). Reliability, robustness, and reproducibility in mouse behavioral phenotyping: A cross-laboratory study. Physiol. Genom..

[18] Mead RJ (2011). Optimised and rapid pre-clinical screening in the SOD1G93A transgenic mouse model of amyotrophic lateral sclerosis (ALS). PLoS One.

[19] Lewis KE (2014). Microglia and motor neurons during disease progression in the SOD1G93A mouse model of amyotrophic lateral sclerosis: Changes in arginase1 and inducible nitric oxide synthase. J. Neuroinflamm..

[20] Haulcomb MM (2017). Locomotor analysis identifies early compensatory changes during disease progression and subgroup classification in a mouse model of amyotrophic lateral sclerosis. Neural Regen. Res..

[21] Alves CJ (2011). Early motor and electrophysiological changes in transgenic mouse model of amyotrophic lateral sclerosis and gender differences on clinical outcome. Brain Res..

[22] Estévez-Silva HM (2022). Pridopidine modifies disease phenotype in a SOD1 mouse model of amyotrophic lateral sclerosis. Eur. J. Neurosci..

[23] Vinsant S (2013). Characterization of early pathogenesis in the SOD1G93A mouse model of ALS: Part II, results and discussion. Brain Behav..

[24] Niessen H (2006). In vivo quantification of spinal and bulbar motor neuron degeneration in the G93A-SOD1 transgenic mouse model of ALS by T2 relaxation time and apparent diffusion coefficient. Exp. Neurol..

[25] Hegedus J, Putman C, Gordon T (2007). Time course of preferential motor unit loss in the SOD1 G93A mouse model of amyotrophic lateral sclerosis. Neurobiol. Dis..

[26] Clark RM, Blizzard CA, Young KM, King AE, Dickson TC (2017). Calretinin and neuropeptide Y interneurons are differentially altered in the motor cortex of the SOD1G93A mouse model of ALS. Sci. Rep..

[27] Boillée S, Vande Velde C, Cleveland DW (2006). ALS: A disease of motor neurons and their nonneuronal neighbors. Neuron.

[28] Philips T, Rothstein JD (2014). Glial cells in amyotrophic lateral sclerosis. Exp. Neurol..

[29] Morandell K, Huber D (2017). The role of forelimb motor cortex areas in goal directed action in mice. Sci. Rep..

[30] Jeong M (2021). Interhemispheric cortico-cortical pathway for sequential bimanual movements in mice. eNeuro..

[31] Brown AR, Mitra S, Teskey GC, Boychuk JA (2022). Complex forelimb movements and cortical topography evoked by intracortical microstimulation in male and female mice. Cereb. Cortex.

[32] Dutta K, Thammisetty SS, Boutej H, Bareil C, Julien J-P (2020). Mitigation of ALS pathology by neuron-specific inhibition of nuclear factor kappa B signaling. J. Neurosci..

