# Supplementary material for: A novel assessment of fine-motor function reveals early hindlimb and detectable forelimb deficits in an experimental model of ALS
Source: Sci Rep. 2022 Oct 11;12:17010. doi: 10.1038/s41598-022-20333-1 (PMC9553953; doi:10.1038/s41598-022-20333-1)
Supplement: Supplementary file 1 — Supplementary Information. [file 41598_2022_20333_MOESM1_ESM.pptx]

## Slide 1
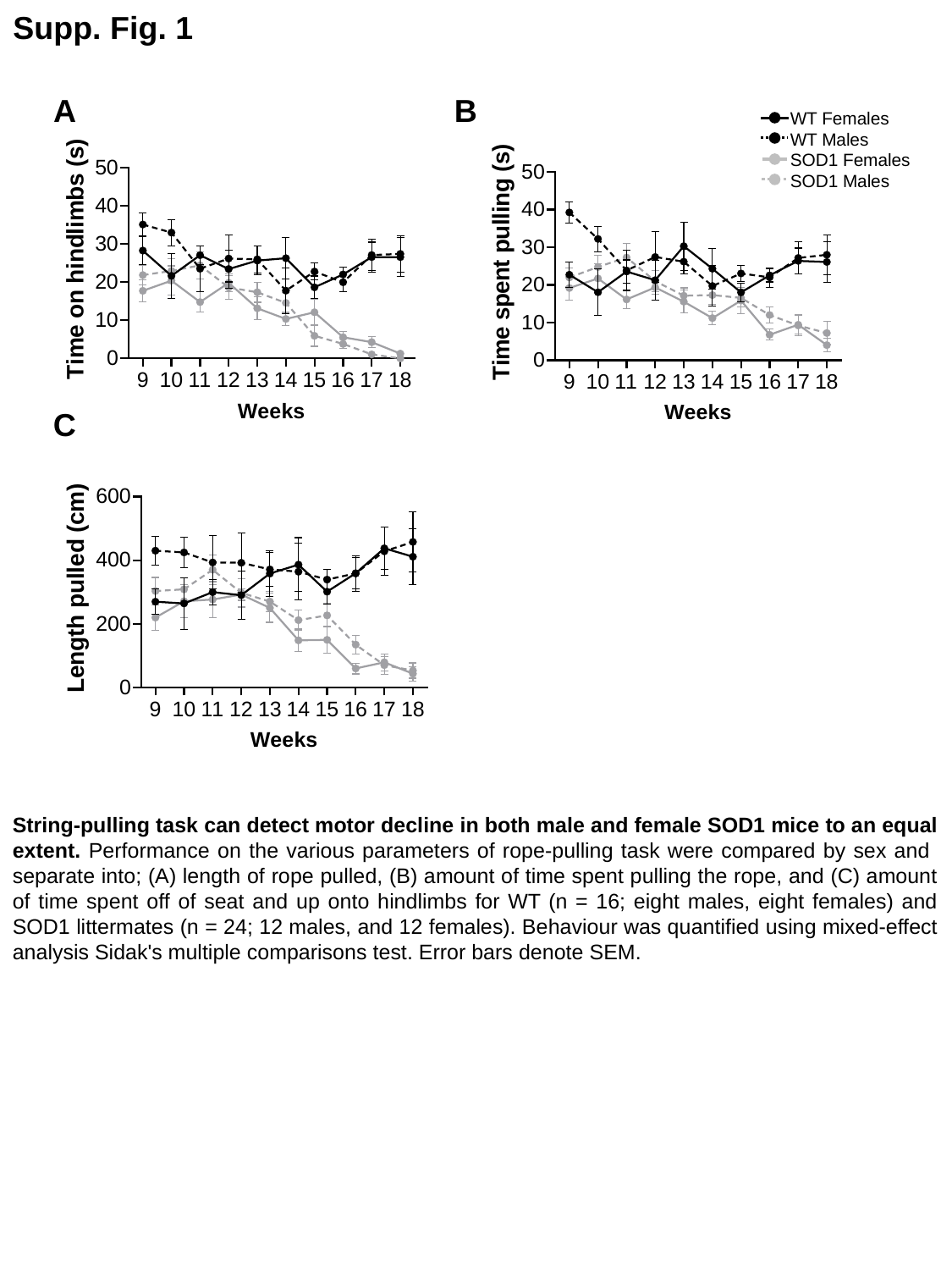

Supp. Fig. 1
A
B
WT Females
WT Males
SOD1 Females
SOD1 Males
C
String-pulling task can detect motor decline in both male and female SOD1 mice to an equal extent. Performance on the various parameters of rope-pulling task were compared by sex and separate into; (A) length of rope pulled, (B) amount of time spent pulling the rope, and (C) amount of time spent off of seat and up onto hindlimbs for WT (n = 16; eight males, eight females) and SOD1 littermates (n = 24; 12 males, and 12 females). Behaviour was quantified using mixed-effect analysis Sidak's multiple comparisons test. Error bars denote SEM.

## Slide 2
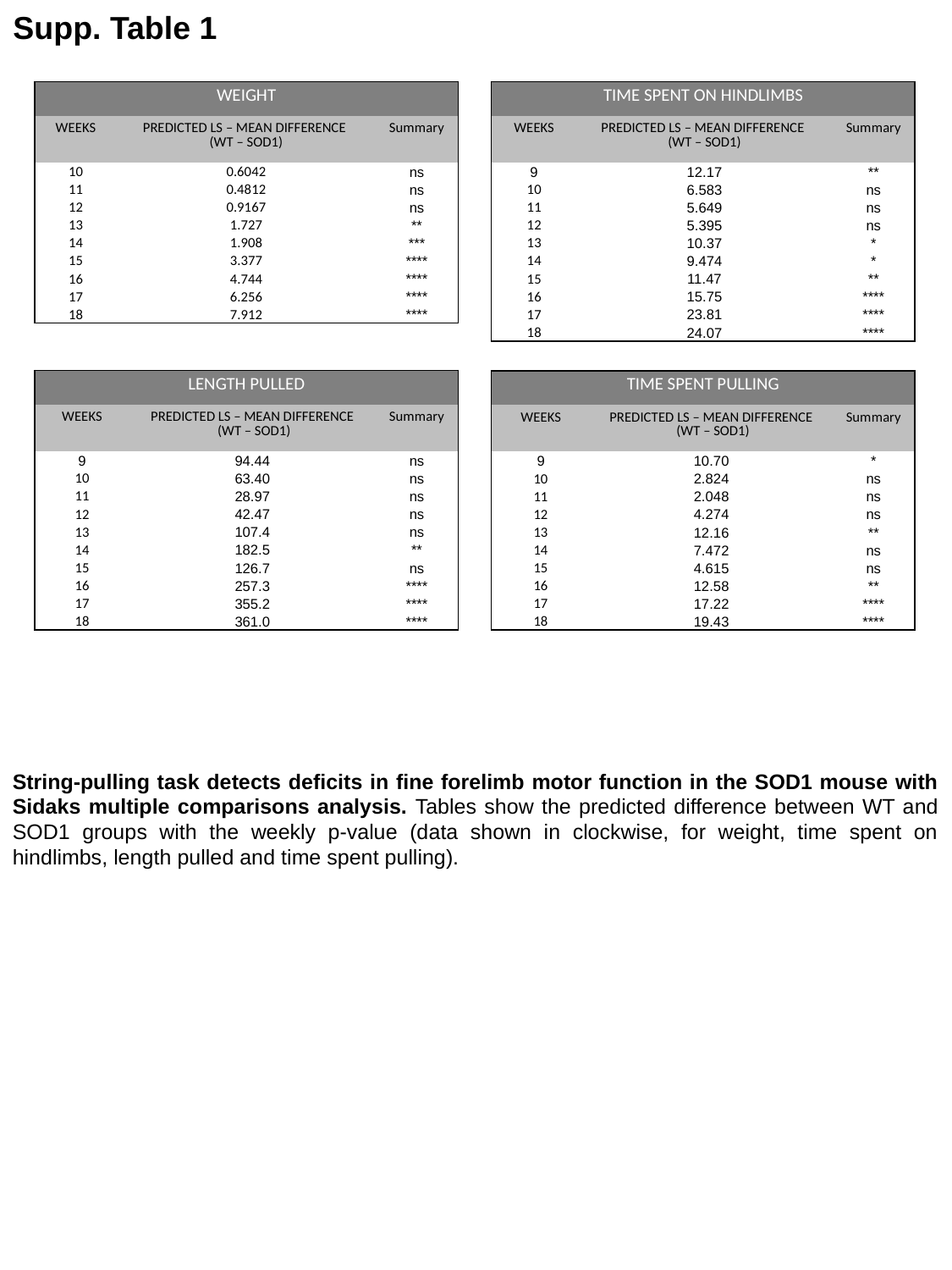

Supp. Table 1
| WEIGHT | | |
| --- | --- | --- |
| WEEKS | PREDICTED LS – MEAN DIFFERENCE (WT – SOD1) | Summary |
| 10 | 0.6042 | ns |
| 11 | 0.4812 | ns |
| 12 | 0.9167 | ns |
| 13 | 1.727 | \*\* |
| 14 | 1.908 | \*\*\* |
| 15 | 3.377 | \*\*\*\* |
| 16 | 4.744 | \*\*\*\* |
| 17 | 6.256 | \*\*\*\* |
| 18 | 7.912 | \*\*\*\* |
| TIME SPENT ON HINDLIMBS | | |
| --- | --- | --- |
| WEEKS | PREDICTED LS – MEAN DIFFERENCE (WT – SOD1) | Summary |
| 9 | 12.17 | \*\* |
| 10 | 6.583 | ns |
| 11 | 5.649 | ns |
| 12 | 5.395 | ns |
| 13 | 10.37 | \* |
| 14 | 9.474 | \* |
| 15 | 11.47 | \*\* |
| 16 | 15.75 | \*\*\*\* |
| 17 | 23.81 | \*\*\*\* |
| 18 | 24.07 | \*\*\*\* |
| LENGTH PULLED | | |
| --- | --- | --- |
| WEEKS | PREDICTED LS – MEAN DIFFERENCE (WT – SOD1) | Summary |
| 9 | 94.44 | ns |
| 10 | 63.40 | ns |
| 11 | 28.97 | ns |
| 12 | 42.47 | ns |
| 13 | 107.4 | ns |
| 14 | 182.5 | \*\* |
| 15 | 126.7 | ns |
| 16 | 257.3 | \*\*\*\* |
| 17 | 355.2 | \*\*\*\* |
| 18 | 361.0 | \*\*\*\* |
| TIME SPENT PULLING | | |
| --- | --- | --- |
| WEEKS | PREDICTED LS – MEAN DIFFERENCE (WT – SOD1) | Summary |
| 9 | 10.70 | \* |
| 10 | 2.824 | ns |
| 11 | 2.048 | ns |
| 12 | 4.274 | ns |
| 13 | 12.16 | \*\* |
| 14 | 7.472 | ns |
| 15 | 4.615 | ns |
| 16 | 12.58 | \*\* |
| 17 | 17.22 | \*\*\*\* |
| 18 | 19.43 | \*\*\*\* |
String-pulling task detects deficits in fine forelimb motor function in the SOD1 mouse with Sidaks multiple comparisons analysis. Tables show the predicted difference between WT and SOD1 groups with the weekly p-value (data shown in clockwise, for weight, time spent on hindlimbs, length pulled and time spent pulling).

## Slide 3
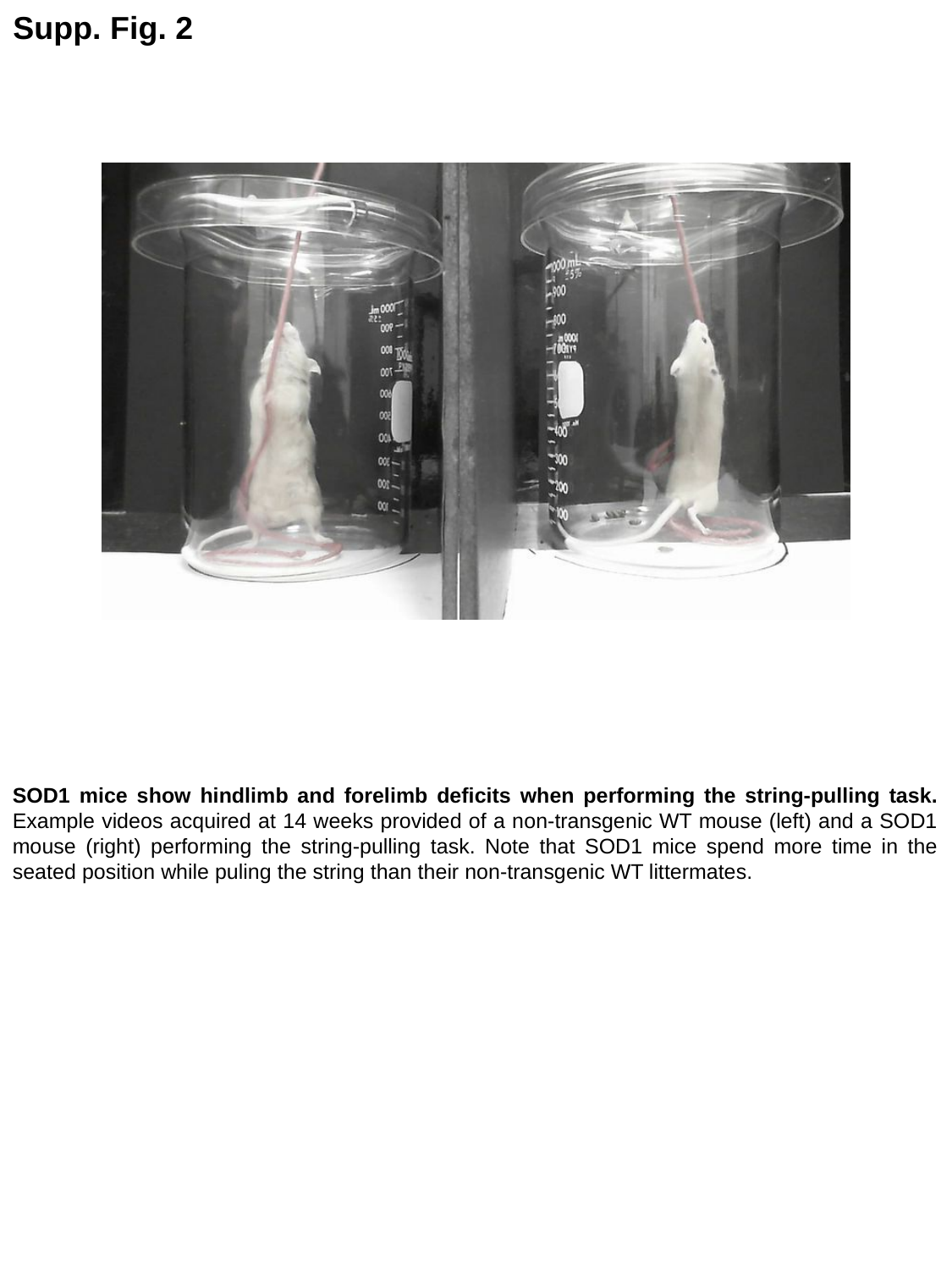

Supp. Fig. 2
SOD1 mice show hindlimb and forelimb deficits when performing the string-pulling task. Example videos acquired at 14 weeks provided of a non-transgenic WT mouse (left) and a SOD1 mouse (right) performing the string-pulling task. Note that SOD1 mice spend more time in the seated position while puling the string than their non-transgenic WT littermates.
